# Revisiting the alpha-synuclein paradox in melanoma-Parkinson’s disease connection: more than a tale of two cell fates

**DOI:** 10.1007/s00018-025-05985-2

**Published:** 2025-12-29

**Authors:** Jacopo Aiello, Roberta Zamarato, Claudia Moscheni, Cristiana Perrotta, Mario Clerici, Daria Trabattoni, Mara Biasin, Fiona Limanaqi

**Affiliations:** 1 Department of Biomedical and Clinical Sciences, University of Milan, Milan, Italy; 2 Fondazione IRCCS Ca’ Granda Ospedale Maggiore Policlinico, Milan, Italy; 3 Department of Pathophysiology and Transplantation, University of Milan, Milan, Italy; 4 IRCCS Fondazione Don Carlo Gnocchi ONLUS, Milan, Italy

**Keywords:** Neurodegeneration, Cancer, Dopamine, Iron, (Neuro)melanin, Oxidative stress, Proteostasis, Autophagy, Inflammation, Major histocompatibility complex

## Abstract

Since the first report in 1972, several studies have documented an association between Parkinson’s disease (PD) and melanoma. Up to 20-fold increased risk of melanoma was reported in PD patients, while a personal/family history of melanoma was linked to a 1.85-fold PD risk. Neurons and melanocytes, which both derive from the neuroectodermal crest, share biological pathways that may be dysregulated in both diseases. In particular, accumulation of the alpha-synuclein (α-syn, SNCA) protein, a pathological hallmark of PD, is also observed in melanoma. Indeed, dysregulated α-syn proteostasis is known to disrupt several biological pathways which can co-incidentally, albeit paradoxically contribute to both neurodegeneration and hyper-proliferative cell growth. These include abnormalities in dopamine (DA), melanin, and iron metabolism, oxidative stress, DNA damage/repair response, inflammation, as well as alterations in mitochondrial function, and cell-clearing machinery. Although α-syn depletion was shown to attenuate melanoma cell proliferation and neurodegeneration, it remains unclear whether α-syn accumulation is a mere culprit of disease, if it represents a common outcome from shared upstream mechanisms, or, finally, a compensatory response to cellular stress. In an effort to elucidate how α-syn bridges melanomagenesis and the neurodegenerative events of PD, this review discusses specific cellular and molecular pathways related to α-syn proteostasis, including environmental factors implicated in melanocytic transformation, such as UV radiation. Addressing open questions and establishing novel experimental models remain essential for developing effective therapeutic approaches to target melanoma and PD without overlooking their comorbidity.

## Introduction

Parkinson’s disease (PD), one of the most prevalent neurodegenerative disorders, is primarily characterized by the progressive loss of dopaminergic neurons in the substantia nigra pars compacta (SNpc), and dopamine (DA) depletion in the striatum. A pathological hallmark of PD is the accumulation of alpha-synuclein (α-syn, SNCA) within intracellular inclusions known as Lewy bodies [1], which has led to the classification of PD within a broader spectrum of neurological disorders named synucleinopathies [2]. α-Syn is a 14 kDa protein with an intrinsically disordered and highly dynamic structure, which makes it prone to misfold and aggregate when mutated or overexpressed [3]. Several point mutations (A30P, E46K, H50Q, G51D, A53E, A53T), as well as duplications and triplications in the *SNCA* gene have been identified that collectively account for < 2% of all PD cases [4, 5]. However, α-syn aggregates are a hallmark of almost all PD phenotypes, suggesting that α-syn is constantly recruited during the natural history of PD independently of the disease trigger. As discussed in previous excellent reviews, both experimental models of PD and post-mortem analyses of PD brains have consistently documented increased levels of α-syn, particularly its aggregated forms [6]. This has led to the consensual view that increased α-syn levels, alterations of its native structure and function, and eventually, its prion-like propagation, represent key and constant steps in the molecular chain of events contributing to neurodegeneration.

**Fig. 1:**
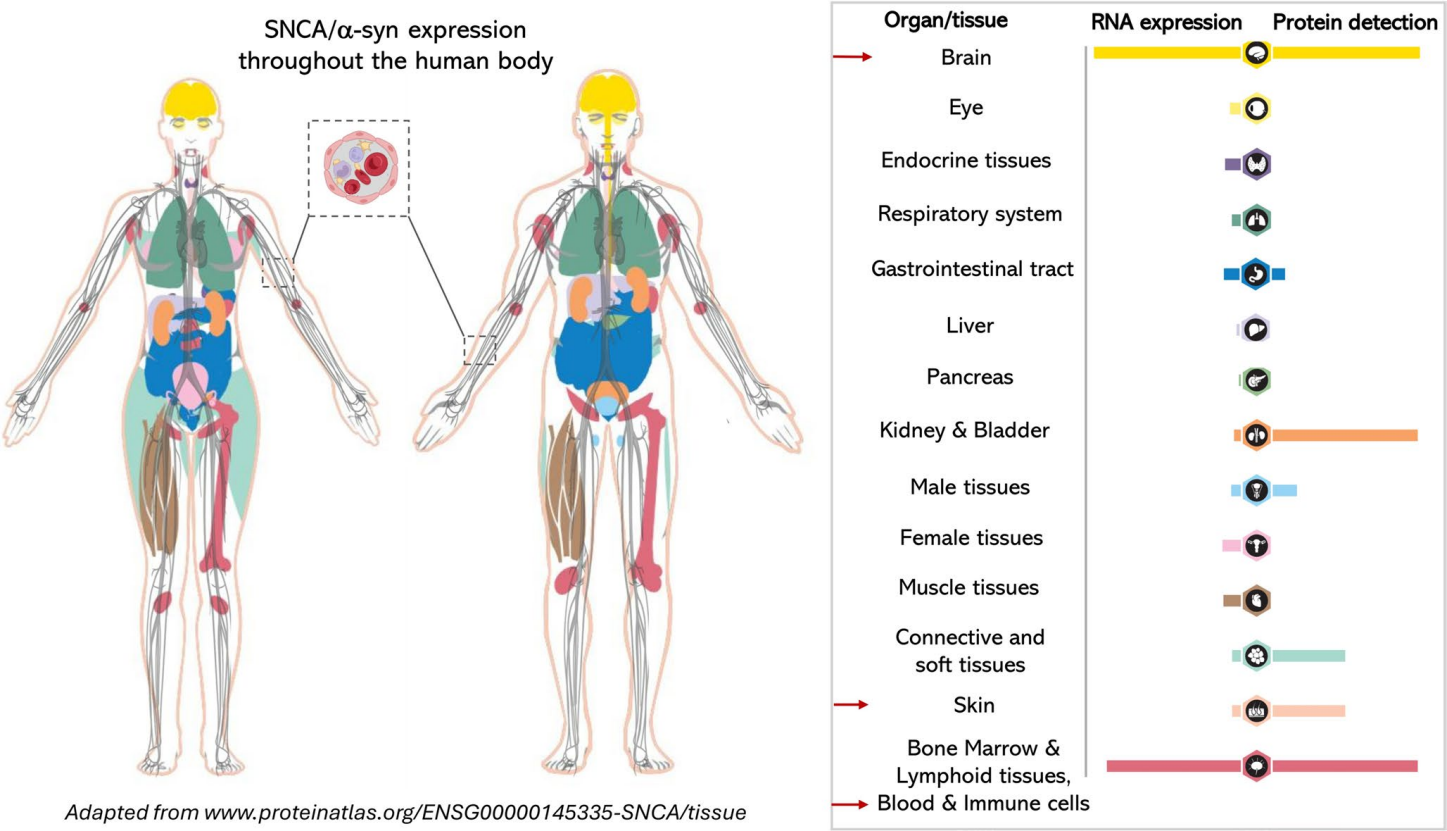
Expression of SNCA/α-syn throughout human tissues. In addition to the central nervous system, SNCA/α-syn is expressed in a variety of non-neuronal tissues and cells, such as skin, muscle, kidney, liver, spleen, lung, bladder, myocardial muscle, endothelial cells/blood vessels, bone marrow, lymphoid tissues, and blood cells, including erythrocytes, megakaryocytes, platelets, T and B lymphocytes, natural killer cells, monocytes and macrophages. In particular, skin and blood cells express high levels of α-syn (red arrows), supporting their utility as non-invasive, diagnostic sources. The presence of α-syn across major tissue systems highlights the multifaceted physiological roles of this protein beyond the brain, suggesting broader relevance to systemic processes and potential pathogenic implications. The figure was modified from www.proteinatlas.org/ENSG00000145335-SNCA/tissue

The expression of α-syn, both at RNA and protein levels has been detected in a plethora of tissues and cell types beyond neurons [7–11] (Fig. 1, www.proteinatlas.com).

In line with this, the accumulation of α-syn in peripheral tissues has emerged as a potential diagnostic tool for PD [12–14]. In particular, increased/aggregated α-syn has been detected within various skin cell types from PD patients, including fibroblasts, keratinocytes and melanocytes [15–19]. This is noteworthy, given the bulk of literature documenting epidemiological and molecular connections between PD and melanoma, one of the most serious and deadly forms of skin cancer [20]. Features that characterize cancer cells, including unrestricted cell growth, and resistance to apoptosis, stand in stark contrast to the progressive neuronal degeneration that characterizes PD, portraying two opposite pathological conditions. In this scenario, melanoma stands as an exception, as it shows a remarkably high co-occurrence with PD.

Since the first report in 1972 about the unusual development of multiple melanomas in a PD patient [21], which was followed by a 30-year-long gap on the topic, a string of data has validated the link between the two diseases. In detail, melanoma incidence among individuals with PD ranges from 1.4– to 20-fold compared to healthy subjects [22]. Initially, this was attributed to L-DOPA, a common treatment for PD, and a precursor for both DA and melanin synthesis in DAergic neurons and melanocytes. However, subsequent studies showed that higher rates of melanoma are present in PD patients independently of L-DOPA treatment [23, 24]. The bidirectionality of this link was confirmed by the observation that melanoma patients also exhibit a 1.85-fold greater risk of developing PD compared to those without a personal/family history of melanoma [25].

A biological link between PD and melanoma is supported by the shared neuroectodermal origin of neurons and melanocytes, which despite having distinct functions, rely on overlapping biological pathways that can be disrupted in both diseases. Two major molecular intersections have been identified, namely DA/melanin metabolism, and α-syn accumulation, both of which may contribute to the co-occurrence of PD and melanoma [22]. Firstly, L-DOPA and DA, the essential precursors in melanogenesis, are enzymatically involved in processes that are typically dysregulated in melanoma; at the same time, PD is characterized by degeneration of neuro(melanin)-containing DAergic neurons. In addition, α-syn accumulation is present in melanoma besides PD [26]. Interestingly, these two processes are not independent events, as α-syn is known to modulate DA and melanin metabolism by acting on tyrosine hydroxylase (TH) and tyrosinase (TYR), two key enzymes in DA and melanin synthesis. Along these lines, it is noteworthy that several key proteins which are modulated by or interact with α-syn, are expressed in both neuronal and melanocytic lineages. This is the case of the cell adhesion molecule L1CAM, which orchestrates neuronal axonal growth and guidance, including that of midbrain DA neurons [27, 28], while also promoting melanoma cell mobility, invasion and migration [29, 30]. Recent findings also indicate that α-syn is associated with L1CAM-positive extracellular vesicles that are abundantly detectable in circulating biofluids of PD patients [31]. These observations suggests that the PD–melanoma connection may involve not only shared neuroectodermal and metabolic pathways, but also common extracellular and immune communication routes related to α-syn-linked vesicular trafficking, and L1CAM-dependent intercellular signaling. Collectively, these dual regulatory and communicative roles of α-syn provide a compelling mechanistic bridge between neurodegeneration and melanocytic transformation, reinforcing the biological relationship between PD and melanoma [32].

It is nevertheless unclear how the very same alteration of α-syn could drive two seemingly opposite pathological events: neuronal cell death in PD, and abnormal cell proliferation in melanoma. Likewise, it remains unclear whether α-syn accumulation is a generalized, by-stander feature of melanoma, or whether it plays a functional role in the early biological events that characterize melanoma onset and progression. Addressing these questions also calls for an analysis of the pleiotropic, often cell- and context-specific roles of α-syn within melanocytes, neurons, and a variety of other cell types where α-syn is expressed. Within this conceptual framework, it is conceivable that abnormal α-syn expression and/or loss of its proteostasis (e.g., phosphorylation, aggregation) intermingle with a cascade of molecular, cellular and systemic alterations at the crossroad of PD and melanoma.

This review aims to offer new insights into the pathophysiological connection between PD and melanoma through an integrated perspective on the paradoxical role of α-syn, with a focus on key cellular and molecular pathways implicated in α-syn proteostasis. The reappraisal of available evidence calls for a careful consideration of whether α-syn depletion may be an appropriate therapeutic strategy, suggesting instead an urgent need for identifying upstream pathways that can contribute to fine-tuning α-syn levels and metabolism while preserving its normal cellular, and multisystemic functions.

## α-Synuclein in the skin: expression and functions in melanocytes vs. melanin-containing dopaminergic neurons

### An overview of melanocyte biology

Skin pigmentation is a highly complex and finely regulated process involving specialized skin cells called melanocytes. These cells originate from melanoblasts (MBrcs), non-pigmented precursors that are derived, in turn, from the embryonic neural crest, from which they migrate mostly along the dorsolateral pathway. Melanocytes produce melanin, the pigment responsible for hair and skin coloration. Melanin synthesis requires a highly coordinated series of metabolic pathways that cross multiple intracellular compartments and organelles, as detailed by previous reviews [33].

Melanin is synthesized and stored in melanosomes, specialized intracellular organelles that play a crucial role in protecting the skin from the harmful effects of Ultraviolet (UV) radiation. Once mature, melanosomes are rerouted from perinuclear to more peripheral cellular regions via a specific transport system, including cytoskeleton proteins, as well as the Ras-related protein RAB-27 A (RAB-27 A). In particular, they accumulate at the level of the distal tips of dendritic processes [34]. Key markers of melanin biosynthesis include tyrosinase (TYR), tyrosinase-related protein 1 (TYRP1) and DOPAchrome tautomerase (DCT, also known as tyrosinase-related protein 2, TRP2), whose expression levels are modulated by microphthalmia-associated transcription factor (MITF) [35].

The function and survival of melanocytes is regulated by an intricate network of paracrine factors synthesized mainly by epidermal keratinocytes and dermal fibroblasts [36]. A symbiotic relationship exists between melanocytes and keratinocytes on the one hand, and between melanocytes and dermal fibroblasts on the other. In particular, the transfer of melanin-containing melanosomes from melanocytes to keratinocytes represents a key mechanism that confers even skin pigmentation and photoprotection. Although great progresses have been made in understanding melanin biosynthesis and intracellular trafficking, the processes involved in melanin secretion and uptake by neighboring keratinocytes remain a topic of ongoing investigation. Transmission electron microscopy studies have provided valuable insights, documenting the fusion of melanosomal vesicles with the plasma membrane, followed by the release of the melanin pigment — referred to as melanocore or naked melanin — into the extracellular space. After secretion, the melanocore is internalized by surrounding keratinocytes [34], though alternative mechanisms for melanin transport and release beyond exocytosis–endocytosis have been postulated, including the involvement of membrane-bound tubules, membrane fusion and cytophagocytosis [37].

### α-Syn in melanocytes

The detection of α-syn in normal human melanocytes raised the question of whether this protein is involved in melanin pigment production, menalocyte homeostasis, and consequently, melanocytic transformation [18, 38]. By acting as a downstream target of MITF, a central regulator of melanocyte development that also controls genes implicated in pigmentation (e.g., TYR, TYRP1, and TRP2), α-syn shows higher expression in differentiated melanocytes compared with MBrcs, suggesting that α-syn is normally upregulated during melanocyte maturation [38].

Functional SNCA-KO studies in experimental models showed that, despite being a downstream target of MITF, α-syn does not grossly alter intracellular melanin content and skin pigmentation, at least under unperturbed homeostatic conditions [38]. Instead, upon UVB exposure specifically, α-syn overexpression was found to reduce melanin synthesis and content in melanoma cell lines [32]. This notwithstanding, α-syn was shown to localize within melanosomes and influence both their morphology and extracellular melanin release [38, 39]. Indeed SNCA-KO in melanocytes leads to a higher amount of dendritic spreading, as well as longer and thinner dendrites compared with control cells [38]. This is accompanied by decreased transport of mature melanosomes to the dendritic tips, as well as reduced melanosome release and transfer to keratinocytes, suggesting a key role of α-syn in melanosome trafficking and secretion [38].

**Fig. 2:**
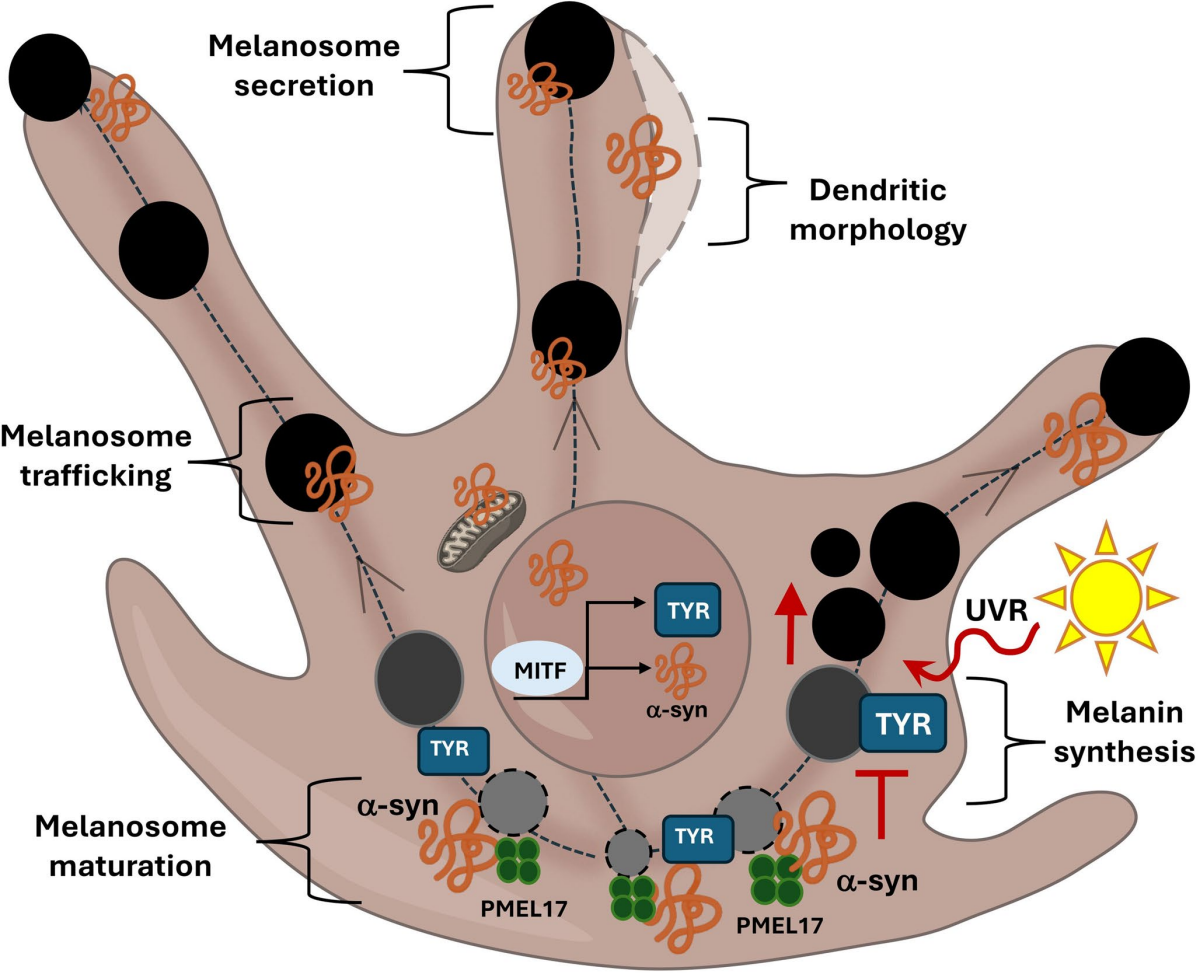
α-Syn localization and functions in melanocytes. α-Syn is transcriptionally regulated by MITF, the master transcriptional factor for melanocyte development that promotes increasing α-syn expression during melanocyte differentiation. Within melanocytes, α-syn colocalizes with melanosomes and is involved in their maturation via interaction with PMEL17, their trafficking to the dendritic tips, and eventually their extracellular release towards skin keratinocytes. Again, α-syn is implicated in shaping melanocytic dendrite morphology. While α-syn does not impact melanin synthesis at baseline, it counteracts UVB-induced TYR upregulation to tone down melanin synthesis. Finally, α-syn is also expressed in the nucleus and melanocytic mitochondria, with no apparent changes in mitochondrial density or distribution

These results are consistent with the validated role of α-syn in modulating synaptic vesicle trafficking, neurotransmitter release, and potentially, neurite morphology via interaction with cytoskeleton proteins within neuronal cells [40–42]. It should be noted that no direct effect of α-syn on the RAB27A complex, a key component in the intracellular transport mechanism of melanosomes, has been observed to date. This suggests that α-syn could affect the transport of melanosomal vesicles by engaging alternative routes, such as the SNARE accessory protein alpha-SNAP, analogously to its role in synaptic vesicle fusion and release in neurons [38]. Although limited, these results indicate a novel physiological role of α-syn in melanocytic cell morphology and its possible involvement in pigment secretion in melanocytes, paving the way for more in-depth studies aimed at elucidating the role of α-syn in this process (Fig. 2).

α-Syn is also expressed in skin keratinocytes and fibroblasts, though its specific roles in these cell types remain poorly explored compared with melanocytes. The few available studies focus on pathological/excess α-syn, with results comparable to those observed in neurons rather than melanocytes. For instance, human skin fibroblasts from PD patients feature decreased cell growth, impaired mitochondrial function, and increased sensitivity to the nigrostrial toxicant paraquat compared to healthy controls [43]. This is in line with studies showing that exogenously added α-syn can be taken up by skin fibroblasts, leading to impaired mitochondrial function, and cell death [44]. Likewise, exogenously added α-syn oligomers were documented to induce degeneration of reconstructed human epidermis by diminishing keratinocyte proliferation while increasing NF-kB nuclear translocation and inflammation in keratinocytes [45].

Similar to what observed in neurons and other skin cells, α-syn was also identified within mitochondria of melanocytic cells, through no significant changes in the quantity or distribution of mitochondria have been observed following SNCA-KO [38]. This suggests that α-syn has cell-type-specific functions, or that it is regulated differently in melanocytes compared with other skin cells and neurons.

### The apparent paradox of α-syn in melanin synthesis within melanocyes vs. DAergic neurons

Worthy of note is the controversial role of α-syn depending on cell type, expression levels, and environmental context (e.g. baseline vs. stressful, disease-relevant conditions). On the one hand, α-syn ablation does not appear to affect basal melanin synthesis in normal melanocytes, suggesting that it is dispensable for baseline pigment production [38]. On the other hand, α-syn overexpression was shown to prevent UVB light-induced increase of melanin synthesis in melanoma cell lines [32]. This was associated with a reduction of TYR activity compared to non-α-syn-expressing cells, suggesting that during stressful conditions (e.g. melanoma-promoting events), α-syn negatively regulates the melanin synthesis response.

In contrast, in DAergic SH-SY5Y neuronal cells, and PC12 neuronal-like cells, α-syn overexpression was shown to increase melanin content [32]. Mechanistically, α-syn is known to inhibit TH activity in DAergic neurons at baseline, while a loss of normal α-syn function due to its accumulation/aggregation leads to decreased DA release and increased levels of intracellular DA [3]. Subsequent DA convertion into neuro(melanin) and reactive quinones may eventually contribute to enhancing the susceptibility to oxidative stress-induced neuronal injury relevant to PD.

A key element that may reconcile these apparently opposite effects lies in both the intrinsic proliferative potential of the cells involved, and the structural plasticity of α-syn itself. In rapidly dividing cells, soluble, monomeric α-syn, generally considered non-toxic, can exert pro-survival and regulatory effects, while the ongoing cell turnover allows the dilution of any potentially harmful, excess, or misfolded species. In contrast, in post-mitotic DAergic neurons, where cellular division cannot dilute exess or toxic conformations, α-syn overexpression or DA-metabolism-related oxidative stress promotes its aggregation into β-sheet–rich, pathogenic forms, leading to cellular dysfunction and death. Thus, the paradox rather reflects protein-intrinsic, and cell-type–specific capacities for proteostasis and renewal, which together determine whether α-syn acts as a physiological modulator or a pathological agent.

#### Modelling α-syn and (neuro)melanin interplay: lessons from Parkinson’s disease

The apparently paradoxical role α-syn in melanin synthesis in melanocytes vs. neurons mirrors the dual role of melanin in melanoma and DAergic neurons. Neuromelanin, a dark, insoluble pigment found within specific populations of DAergic and noradrenergic neurons (SNpc, and Locus Coeruleus), can act as an antioxidant and neuroprotectant by binding toxic metabolites such as free iron, oxidized DA, DA metabolites and metals, as well proteinaceous oxidized/aggregates, including α-syn itself [46–48]. However, neuromelanin deposition can also make neurons more sensitive to DA-dependent oxidative stress, overwhelmed cell-clearance capacity, α-syn aggregation, and α-syn-related toxicity [49, 50]. This is supported by evidence that α-syn accumulation is exacerbated in melanized DAergic neurons [51], and α-syn aggregates preferentially redistribute to neuromelanin granules in the SNpc early in PD but not in healthy controls [52, 53].

Recent advances in the context of PD have greatly expanded our ability to model the interaction between α-syn and neuromelanin. A growing body of work, particularly from Vila and colleagues, has demonstrated that neuromelanin is not merely a bystander pigment, but an active determinant of DAergic vulnerability and α-syn aggregation [50, 54–58]. A major conceptual and technical advance came from studies in which human TYR was overexpressed in the rodent brain to induce age-dependent neuromelanin biosynthesis, thereby addressing a long-standing gap in the field, namely the natural absence of neuromelanin within rodent catecholaminergic neurons [54, 58]. Remarkably, these animals develop progressive DAergic neuron loss accompanied by α-syn-positive inclusions, which extend to other midbrain catecholaminergic populations involved in autonomic regulations, providing the first direct evidence that neuromelanin production itself can initiate PD-like pathology [54, 58]. Most notably, while neuromelanin accumulation above a pathogenic threshold is sufficient to drive PD-like pathology, including the occurrence of α-syn-positive inclusions, α-syn itself is not actually required for neither inclusion formation nor nigrostriatal neurodegeneration, as SNCA-KO did not rescue DAergic cell loss or striatal DA denervation in these animals [54]. Instead, maintaining intracellular neuromelanin levels below their pathogenic threshold was later shown to succesfully mitigate in vivo parkinsonian features [55]. This was achieved through overexpression of the vesicular monoamine transporter 2 (VMAT2) reducing the pool of potentially toxic oxidized DA species that serve as precursors for neuromelanin formation.

In non-human primates, similar to what oberserved in rodents, time-dependent neuromelanin buildup achieved through human TYR overexpression, drives endogenous synucleinopathy and neuronal loss [56], firmly linking pigment accumulation to PD pathogenesis. Again, hyperactive microglial cells bearing the PD-realted LRRK2 mutation exposed to neuromelanin were shown to induce selective DAergic neurodegeneration [57]. Interestingly, such an effect was not ricapitulated by exposure to α-syn fibrils and it was instead prevented by pre-treatment with the immunomodulatory compound ivermectin, undersocring the neuromelanin-immune axis as a key contributor to DAergic vulnerability in PD [57]. Consistently, post-mortem midbrain tissue from LRRK2-PD patients revealed pronounced microglial activation surrounding neuromelanin-laden neurons, corroborating the in vitro findings [57]. Complementary imaging studies in humans and rat models further reveal longitudinal neuromelanin dynamics in prodromal and early PD, whereby neuromelanin-sensitive MRI displays a biphasic pattern: an initial signal increase reflecting neuromelanin accumulation in DAergic neurons, followed by a decrease associated with neurodegeneration [59]. Such experimental systems are instrumental for understanding how pigment overload and disrupted proteostasis converge to determine neuronal vulnerability, bridging molecular mechanisms to clinical manifestations of disease.

Collectively, it might be inferred that, while native α-syn regulates DA and melanin metabolism, excess neuromelanin acts a major upstream driver of α-syn aggregation, oxidative stress, and neuroinflammation. Strikingly, many of these processes are profoundly dysregulated in melanoma as well. Melanin also has a dual role in skin biology: while it effectively absorbs and dissipates UVB radiation, reducing direct DNA damage and thereby lowering the risk of UVB-induced skin cancers, melanin may also become a photosensitizer. This is particularly evident under UVA exposure that leads to oxidative stress and DNA damage, potentially increasing the risk of melanoma [60]. As we shall see in the next paragraph, α-syn accumulation is a hallmark of melanoma, a condition associated with reduced melanin synthesis and a spectrum of cellular events implicated in melanocytic transformation.

## α-Syn in melanoma

Melanoma, characterized by uncontrolled growth of skin melanocytes, is one of the most aggressive forms of cancer, with a rising global incidence of 3–5 per 100,000 patients, and a very high mortality rate (65% of skin cancer-related deaths) [61]. The increasing evidence about α-syn accumulation in melanoma has positioned this protein as the most compelling molecular bridge between PD and melanoma, also reinforcing the biological relevance of their co-morbidity [22, 26]. In detail, skin tissue, mostly melanocytes, but also fibroblasts and keratinocytes from melanoma patients, as PD ones, feature increased α-syn levels compared with healthy controls [18, 26]. Furthermore, a ~ 3-fold higher SNCA expression is reported in metastatic growth phase melanomas compared to healthy controls [62], suggesting that α-syn accumulation in the skin may not merely reflect propagation along neural routes, but rather arise from cell-intrinsic dysregulation within melanocytic or tumorigenic contexts. Finally, a very recent study conducted on skin biopsies from both PD and melanoma patients documented an abnormal redistribution of α-syn from the nuclei to the cytoplasm [63].

An increasing body of literature has been focused on elucidating the relationship between α-syn and melanoma. Despite consistent evidence for α-syn accumulation in melanoma cells across different studies, the biochemical nature of the accumulated protein remains unclear, as most reports detected total α-syn, though phosphorylated/oligomeric α-syn species were reported sporadically [62, 64–66]. Endogenous α-syn levels vary markedly among different cell lines under identical experimental conditions [22, 62, 64]. Increasing research efforts have focused on manipulating α-syn expression in melanoma, following experimental approaches analogous to those employed for studying α-syn in the PD-like brain, as summarized in Table 1. Functional studies of SNCA overexpression showed that α-syn enhances cellular proliferation in melanoma cell lines [67]. Again, SNCA-A53T mutant mice xenografted with melanoma cells show enhanced tumorigenesis compared to WT mice [67]. Along the same lines, numerous SNCA ablation studies have provided valuable insights into various α-syn-related processes in melanoma, as dissected in the following subsections.

**Table 1:** Summary of major α-syn-manipulating experimental models in melanocytes, melanoma, and neuronal/dopaminergic systems

	Model/Cell Type	Manipulation	Observed Effect	Interpretation/Mechanistic Insight	Ref.
Melanocytes and Melanoma	Normal human melanocytes	SNCA-KO	No significant effects on basal melanin synthesis ↑Dendrite length/number **↓**Transport of mature melanosomes to dendritic tips ↓Melanosome release/transfer to keratinocytes	α-Syn is dispensable for baseline pigmentation. α-Syn supports melanosome maturation/trafficking and secretion.	[38]
	Melanoma cell lines (B16, A375, SK-MEL-28)	SNCA overexpression	↓TYR expression/activity and melanin synthesis under UVB ↑Cell proliferation, migration/invasion	α-Syn represses UVB-induced melanogenesis. α-Syn enhances oncogenic and pro-migratory behavior.	[32, 67]
	Human melanoma cell line (SK-MEL-28)	SNCA-KO	**↓**Cell motility/invasion/migration ↓Anterograde transport of endolysosomes ↓ L1CAM, CD81, N-cadherin, TfR1 ↑Endolysosomal buildup and degradation of adhesion proteins	α-Syn is required for anterograde trafficking and plasma membrane recycling of adhesion proteins fostering invasive phenotypes. α-Syn promotes pro-migratory tetraspanin pathways.	[29, 30, 68]
			↑Inflammatory/immune genes (e.g., IL1B, SAA1, CXCL8, CXCL10; matrisome factors)	α-Syn may aid immune evasion by suppressing immune-activating factors.	[69]
	Murine SCID ◊ SNCA-KO melanoma cell SK-MEL-28 xenograft		↓Cell proliferation and tumor growth ↑Apoptosis of tumor xenografts ↓TfR1, ↑ferritin, ↑ROS	α-Syn promotes iron uptake via TfR1 but simultaneously protects against oxidative stress to fuel tumor cell growth.	[70]
	Primary human melanoma cells & melanoma cell lines (SK-MEL-28, A375)		↑Nucleolar DNA damage ↑Recovery from DSB-induced damage	α-Syn promotes the nucleolar recruitment of DSB-repair proteins, reducing micronuclei formation.	[71]
	Murine ◊ B16 melanoma cell xenografts	SNCA-A53T overexpression	↑Tumor growth and aggressiveness	α-Syn mutants promote melanoma tumorigenesis.	[67]
		Local tumor SNCA-KO *and/or* systemic host SNCA-KO	↓Tumor growth Systemic KO – ↑anti-tumor immunity Local KO – ↓tumor inflammation	α-Syn exerts distinct systemic vs. tumor-intrinsic immune mechanisms.	[72]
	Human melanoma cell lines WM983-A, WM983-B, SK-MEL-5, WM852, WM1158 & Murine ◊ WM983-B human melanoma cell xenografts	Anti-α-syn oligomerizing compound anle138b	↑Autophagy ↑Melanoma cell death ↓Melanoma cell markers ↓Adhesion molecules and proteins of the oxidative stress/degradation pathway ↑MHC-II	α-Syn promotes melanoma aggressiveness through dysregulation of autophagy. α-Syn may aid immune evasion and tumor progression by impairing lysosomal degradation products presented on MHC class II molecules.	[62, 65]
	Murine ◊ TG3 spontaneous melanoma	SNCA-KO	↓Tumor onset and growth ↓DNA damage ↑Apoptotic markers	α-Syn supports melanoma survival via DNA-damage tolerance, promoting genomic stability.	[73]
Neuronal systems and PD models	Human DA neurons (LUHMES)	SNCA-KO	↓Expression of 401 genes involved in cell cycle regulation, cell proliferation, neuronal differentiation, and synaptic activity	Functional α-syn is required for the expression of cell cycle and differentiation genes.	[74]
	SH-SY5Y and PC12 neuronal-like cells	SNCA overexpression	↑Melanin synthesis	α-Syn enhances DA oxidation and neuromelanin synthesis in neuronal cells.	[32]
	Murine ◊ Thy1-promoter-driven SNCA	Human WT and Mutant A30P/A35T SNCA overexpression	↑Age-dependent neurodegeneration	Human mutant α-syn produces PD-like neuropathology.	[75]
	Murine ◊ CSPα-KO	Human WT or Mutant A35T SNCA overexpression	↓Lethality of CSPα KO mice ↓Neuronal cell death and brain gliosis ↓Motor impairment ↑SNARE complex assembly by WT SNCA	α-Syn acts as a cochaperone to promote neuronal survival.	[75]
		SNCA-KO	↑Lethality of CSPα KO mice ↑Neuronal cell death and brain gliosis ↑Motor impairment ↓SNARE complex assembly by WT SNCA		
	Murine	SNCA-KO	↓Ferritin and transferrin-bound iron (Tf-Fe) in the neuroretina, brain, and hematopoietic organs	α-Syn contributes to the uptake of Tf-Fe by modulating the endocytosis and recycling of Tf/TfR complex	[76]
	S. cerevisiae C. elegans	Human SNCA overexpression	↑Age-dependent degeneration of DAergic neurons ↓Retromer-mediated endocytic recycling of the high-affinity iron importer Fet3/Ftr1	α-Syn dysregulates Fe homeostasis to promote neurodegeneration	[77]
	Rodent ◊ AAV-hTyr-injection in the SNpc	SNCA-KO in neuromelanin-producing mice	No rescue of DA neuron loss or striatal DA depletion	α-Syn is not required for neuromelanin-induced neurodegeneration.	[54]
	Murine	SNCA overexpression	↑CCR2 + monocyte entry from the periphery to the CNS ↑Activation of resident microglia ↑MHC-II expression	α-Syn promotes infiltration of reactive peripheral monocytes to the brain.	[78]
	Murine	SNCA-RNAi	↑DAergic neurodegeneration and nigrostriatal denervation ↑Reactive microglia ↑Neuronal MHC-I	Loss of α-syn within DAergic neurons promotes neuroinflammation and neurodegeneration.	[79]
	iPSC-derived LRRK2-mutant microglia	Exposure to α-syn fibrils or neuromelanin	Microglia exposed to neuromelanin but not α-syn fibrils ◊ ↑ degeneration of co-cultured DAergic neurons	α-Syn is not required for microglial-mediated neurodegeneration in LRRK2-related PD pathology.	[57]
	Primary murine microglia & Human peripheral blood mononuclear cells (PBMCs)	Exposure to WT/modified/mutant α-syn, or neuromelanin	Similar to neuromelanin, α-syn ◊ ↑IFN-γ secretion ◊ ↑ neuronal MHC-I expression in neurons α-syn peptides ◊ antigenic T cell epitopes	α-Syn primes MHC upregulation and T-cell mediated cell death.	[80, 81]
	Murine and human cortical neurons with induced DNA damage & Mouse ◊ α-Syn-GFP	SNCA-KO	↑ Levels of DSBs ↓Non-homologous end-joining forms of DSB repair	α-Syn translocation to the nucleus regulates neuronal repair responses to induced DSBs	[82]
		SNCA reintroduction	↑ α-Syn localization to DSBs ↓Levels of DSBs ↑ Recovery from DSB damage		
	Mouse ◊ α-Syn-GFP	Cortical injection of α-syn preformed fibrils	↓ α-Syn localization to DSBs ↑ Levels of DSBs especially in inclusion-bearing cells	Lewy inclusion-induced loss of nuclear α-Syn disrupts DSB repair	

### Regulation of TYR activity and melanin synthesis

α-Syn is known to reduce the expression and activity of the catalytic enzymes involved in DA and melanin biosynthesis, namely TH and TYR, through the activation of protein phosphatase PP2A, the enzyme responsible for dephosphorylating and inactivating both. While α-syn overexpression was shown to reduce UVB-induced melanin synthesis via TYR downregulation in A375 and SK-MEL-28 melanoma cells, SNCA-KO was shown to increase TYR activity instead, suggesting that α-syn accumulation might contribute to reduced melanin synthesis, a common feature observed in melanoma [32]. However, the inherently rapid proteasomal degradation of TYR in SK-MEL-28 cells led to postulate that α-syn could modulate pigmentation through alternative mechanisms [39]. In fact, α-syn was shown to localize to the melanosome to modulate functional Pmel17 amyloid formation, an important step in melanogenesis, which in turn, may alter melanin production leading to the amelanotic phenotype observed in melanoma [39]. However, the question remains whether the modulation of Pmel17 aggregation by α-syn directly translates to an impact on melanin synthesis. Again, it remains unclear whether α-syn within melanosomes exists in free/native monomeric or aggregated form, highlighting the need for further studies to clarify its biochemical nature.

### Immune evasion and MHC regulation

The immune-modulatory functions of α-syn apply to a variety of cell types and tissues, both in the CNS and periphery. α-Syn normally promotes protective immune reactions against infections [83], partly by supporting interferon signalling [84]. In line with this, deficiency in both T and B lymphocyte development [85, 86] as well as impaired host defence against infectious agents are seen in SNCA-KO mice [87, 88]. In endothelial cells, α-syn ablation promotes age-related dysfunction, hyper-inflammation, and increased blood pressure, which suggests a role of α-syn in sustaining vascular integrity meanwhile toning down inflammation [89, 90]. Remarkably, while upregulation of endogenous α-syn occurs as a prompt response following a variety of immune stimuli [83], α-syn accumulation/aggregation promotes abnormal immune reactions [9, 10, 91, 92], including hyper-inflammation and functional alterations in leukocytes [9, 93–95]. This is key for the pathobiology of PD, since peripheral immune cells from these patients display a hyper-activated and pro-inflammatory profile [96]. Beyond local CNS inflammatory reactions (e.g. microglial activation), peripheral immune cell entry in the CNS has been indeed postulated as a key event for α-syn-induced inflammation and neurodegeneration [78].

This is in line with an increasing number of studies emphasizing the seminal role of both systemic and CNS immune reactions, including the innate and adaptive arms, in the pathophysiology of PD. Along similar lines, research on postmortem human PD brains and animal models showed that α-syn accumulation is accompanied by an exacerbated immune response in the brain, including activation of microglia, release of cytokines and chemokines, exaggerated infiltration of CD4 + T lymphocytes, and accumulation of IgG around degenerated neurons [79, 97, 98]. As previously reviewed, several in vivo and in vitro studies have documented that excess α-syn promotes the activation of the antigen presentation and processing pathway via upregulation of microglial major histocompatibility complex class II (MHC-II) molecules, CD4 + T cells activation, and pro-inflammatory cytokine release [99]. Most importantly, factors released from microglia following activation by neuromelanin or α-syn, or even high cytosolic DA and/or oxidative stress were shown to induce neuronal MHC-I on DA neurons, eventually triggering cell death in the presence of appropriate cytotoxic CD8 + T cells [80]. Thus, in neurons, excess α-syn may also contribute to promoting MHC-I-dependent, auto-immune cell death [81].

Paradoxically, silencing endogenous α-syn also results in a similar pattern of neurodegeneration, namely up-regulation of MHC-1 expression, as well as recruitment of reactive microglia and T-cells to affected DA neurons [100]. Following the induction of neuroinflammation, SNCA-KO results in a 50% loss of DA neurons in the SNpc and a corresponding loss of nigrostriatal terminals and DA concentrations within the striatum [100].

From such evidence it emerges that in both the CNS and systemic milieu, α-syn acts as a double-edged sword: homeostatic at physiological levels to fine-tune the immune response, and pathogenic when misfolded or overexpressed, acting as a danger-associated molecular pattern (DAMP) or as an autoantigen to promote systemic inflammation, neuroinflammation, and neuronal cell death.

Evidence in melanoma also suggests a context-dependent immune-modulatory role of α-syn. A study conducted on mice xenografted with melanoma cells showed that interfering with oligomeric α-syn by using the molecular compound anle138b, leads to a substantial upregulation and expression of MHC-II, potentially promoting anti-melanoma immune responses [65].

Again, by using WT and SNCA-KO B16BL6 melanoma cells xenografted into SNCA-KO and WT mice, it was shown that both systemic and local ablation of SNCA contribute to reduced tumor growth. Interestingly, different underlying mechanisms were observed concerning the immune milieu. In fact, while systemic SNCA ablation increased anti-tumor immune responses, local SNCA ablation decreased tumor inflammatory reactions [72].

Finally, an RNA sequencing analysis of SK-MEL-28 SNCA-KO clones identified several upregulated genes related to the immune system, including the inflammatory response and the matrisome (e.g. IL-1β, SAA1, IGFBP5, CXCL8, and CXCL10), suggesting that high levels of α-syn in melanoma might help the cells evade the immune system by inhibiting the secretion of these immune activating factors [69].

Thus, in the frame of melanoma, α-syn may suppress anti-tumor immunity possibly by modulating MHC-II expression and fostering immune evasion; however, it remains unclear whether tumor-intrinsic α-syn may support a pro-inflammatory, possibly tumor-promoting milieu. These findings underscore the dual, and potentially tissue-specific role of α-syn, which becomes particularly relevant when considering its contribution to PD-melanoma comorbidity. Both PD and melanoma are associated with dysregulated innate and adaptive immunity, raising the compelling question of whether α-syn acts more as a context-sensitive modulator of immune dynamics or rather as a primary pathogenic driver in this interaction.

### Tumor cell motility

α-Syn was shown to be involved in the metastatic process through interaction with CD81 tetraspanin, a pro-migratory and immune-suppressive factor. The levels of expression of these two compounds are strictly correlated; in fact, a recent study on human SK-MEL-28 melanoma cells reported that SNCA-KO leads to a significant decrease in CD81 levels [68]. Specifically, there was a 40% decrease in CD81 mRNA abundance and an 83% decrease in CD81 protein levels across all SNCA-KO clones. This suggests that α-syn regulates CD81 expression at transcriptional, post-transcriptional and post-translational levels [68]. In line with this, α-syn and CD81 have been hypothesized to concertedly play an immunosuppressive role in the melanoma tumor microenvironment, potentially leading to enhanced melanoma aggressiveness and decreased patient survival. While positively correlated between them, SNCA and CD81 were found to be negatively correlated with key cytokine genes involved in immune activation and cell death [68]. This is the case of cytokine-encoding mRNAs associated with immune activation (IL12A, IL12B, IFNG, TNF) and tumor cell elimination (GZMB, PRF1) which, in turn, were found to be significantly lower in melanoma patients with reduced survival [68].

Other studies have found that SNCA-KO causes a reduction of mobility, invasion and migration in SK-MEL-28 melanoma cells compared to control cells expressing α-syn [29]. This was associated with the downregulation of adhesion molecules such as L1CAM and N-cadherin, indicating that α-syn may support melanoma progression by promoting L1CAM and N-cadherin recycling to the plasma membrane [29]. Recent evidence supports a model in which soluble, monomeric α-syn acts as an accessory factor promoting the anterograde trafficking of membrane proteins, including L1CAM, N-Cadherin, CD81, towards the plasma membrane [30]. In SNCA-KO melanoma cells, the absence of α-syn leads to inefficient anterograde transport and redirects these proteins toward lysosomal degradation [30]. Interestingly, this defective transport in SNCA-KO melanoma cells appears to be partially compensated by upregulation of the inflammatory secretory pathway [69]. This trafficking-based interpretation is also consistent with the broader literature implicating monomeric α-syn in vesicular dynamics and membrane protein recycling. Moreover, a similar mechanism might underlie the anterograde transport of melanosomes from melanocytes to keratinocytes, further linking α-syn’s physiological role in vesicle trafficking to pigmentary and tumorigenic processes in the skin.

Overall, these results highlight how α-syn can affect the aggressiveness of melanoma and its evolution, not only by enhancing cellular motility through the transport and trafficking of CD81, L1CAM, and N-cadherin but also by shaping the immune landscape of the tumor microenvironment. Again, these data confirm the intriguing inverse relationship between α-syn, tumor invasion, and immune responses, further puzzling the paradoxical and context-dependent roles of α-syn in melanoma, PD, and PD-melanoma comorbidity.

### Iron homeostasis and oxidative stress

A strong interconnection exists between α-syn, iron, DA/(neuro)melanin metabolism, oxidative stress, and cell clearing systems, which applies to both PD and melanoma.

Iron (Fe) operates in a variety of metabolic processes including DNA synthesis, cell cycle progression, Fe-sulfur cluster biosynthesis, heme synthesis, and energy production. In this regard, cancer cells have a high demand for this metal, and tumors can often subvert normal systemic and cellular Fe homeostasis to acquire high levels of Fe by increasing its uptake and storage while decreasing its export [101]. According to the oxidation status, Fe exists as two main forms, namely Ferric iron Fe (III) and Ferrous iron Fe (II). Fe (III) is poorly soluble, often stored in proteins like ferritin, or bound to transferrin to be transported in the bloodstream. Fe (III) is reduced to Fe (II), which represents the most reactive, soluble and bioactive form, readily participating in redox reactions (e.g., Fenton chemistry, ROS production).

Most importantly, Fe metabolism is key for the homeostasis and redox status of both melanocytes and DA neurons. Though not the central catalytic cofactor in melanin synthesis, Fe is functionally necessary for optimal melanogenesis by influencing melanosome function, and redox balance [33]. In turn, melanin interacts with Fe to impact its metabolism and even overall ROS production. By sequestering reactive metals (including both Fe) to prevent Fenton reactions and additional oxidative damage, melanin acts as a metal ion reservoir for their storage and release [102]. However, melanin also acts as a ferrireductase for Fe III, and it has a higher binding affinity for Fe II, which may become readily oxidized [103]. In fact, both the melanogenic precursor DOPA as well as melanin have been shown to promote the release of reactive Fe (II) depending on the presence of oxygen [33]. Most importantly, increased Fe levels are detected in melanosomes of dysplastic naevi and melanoma compared to healthy melanocytes [104]. Furthermore, Fe (II) is necessary for DA synthesis, as it is a co-factor of TH. Similarly to what observed for melanin, Fe may induce oxidative stress through interaction with neuromelanin [105]. In fact, Fe can catalyse oxidation of DA to quinones, likely contributing to neuromelanin accumulation and greater concentrations of toxic metabolites in neuromelanin granules [106]. Therefore, regulation of the Fe (II)-to-Fe (III) ratio is key for the survival of DA neurons and PD pathogenesis. In line with this, increased brain Fe accumulation is also a feature of PD [107–109].

In this context, α-syn is critically involved in the regulation of Fe metabolism, and in turn, Fe modulates α-syn expression both in the brain and in other tissues. For instance, the expression of α-syn during the different stages of erythropoiesis is closely correlated with that of the major heme metabolism genes such as ALAS2, FECH and BLVRB [110, 111]. Noteworthy, α-syn also acts as a cellular ferrireductase, responsible for reducing Fe III to reactive, bioavailable Fe II [112]. In turn, α-syn has a Fe-responsive element that follows a Fe-mediated expression mechanism [113–115]. While supporting a role for Fe in the translational control of α-syn expression, these data also document a role of Fe chelators in controlling α-syn levels [115]. Finally, α-syn was shown to promote the membrane recruitment of holo-transferrin (Tf)-transferrin receptor 1 (TfR1) complexes, as supported by the occurrence of Fe deficiency and the disruption of TfR1 endocytic recycling to the plasma membrane in a variety of SNCA-KO mouse tissues [76].

In both Saccharomyces cerevisiae and DAergic neurons of Caenorhabditis elegans, α-syn was shown to alter Fe homeostasis by interfering with the high affinity importer Fet3/Ftr1 [77]. Under low external Fe conditions (< 1 µM), Fet3/Ftr1 is maintained on the plasma membrane by retrograde endocytic recycling, whereas it is internalized under high external Fe concentration (>10 µM). Remarkably, both WT and mutated A53T α-syn were shown to phenocopy the high Fe condition, inhibiting the plasma membrane recycling of Fet3/Ftr1. This suggests that α-syn-expressing DAergic neurons are susceptible to abnormal Fe levels, whereby excess Fe enhances α-syn-induced aggregation/toxicity, fueling a vicious cycle which eventually promotes neurodegeneration. In line with this, the iron chelator desferoxamine partly rescues degeneration of DAergic neurons in transgenic worms overexpressing α-syn [77].

As far as melanoma is concerned, a very recent study showed that SNCA-KO in SK-Mel-28 melanoma cells reduces cell proliferation in association with a decrease in TfR1, and a subsequent increase in ferritin, which goes along with ROS accumulation [70]. SNCA-KO SK-Mel-28 xenografted clones also showed reduced tumor growth in mice, which was associated with decreased TfR1, and iron exporter ferroportin (FPN1), along with increased levels of ferritin, the divalent metal ion transporter 1 (DMT1), Fe (III), and apoptosis (TUNEL staining) compared to control melanoma xenografts [70]. This suggests that in melanoma, similarly to DA neurons, α-syn promotes iron uptake, fueling the “iron-seeking phenotype” of tumor cells. However, the final outcomes upon oxidative stress and cell fate may represent a pivotal divergence in the pathogenic mechanisms underlying PD and melanoma. In particular, the observed increase of ROS following SNCA-KO in melanoma suggests that α-syn accumulation may represent a physiological response for this protein to fulfill its intrinsic role, that is to mitigate oxidative stress and melanin synthesis in support of cell survival and proliferation. This stands in stark contrast to PD, where α-syn eventually loses its chaperone-like, natural function [75] and intermingles within a vicious cycle of Fe-driven oxidative damage and neurodegeneration.

Within this framework, it is worthy of note the interaction of α-syn with DJ-1, an oxidative stress regulator that acts as redox-sensitive chaperone and ROS scavenger, but also as an oncogenic protein. Loss-of-function mutations in DJ-1, which are linked to autosomal recessive PD (*PARK7*), are known to compromise a variety of cell functions that are all relevant for α-syn aggregation and toxicity, including oxidative stress, mitochondrial damage, and impaired cell-clearing systems [116]. Intriguingly, a correlation between α-syn and DJ-1 was identified in both primary and metastatic melanoma [66]. In detail, SNCA overexpression in SK-MEL-28 cells fostered DJ-1 upregulation, while temozolomide treatment concurrently reduced both proteins, suggesting that the interaction and/or protein stability of the α-syn-DJ-1 complex could promote melanoma cell survival and proliferation, representing a possible therapeutic target [66].

### Autophagy

α-Syn accumulation/aggregation is known to alter both autophagy-lysosomal and proteasomal degradation in a variety of neuronal and non-neuronal cells [9, 117]. This pathogenic axis—linking Fe overload, ROS production, α-syn aggregation, and impaired cell-clearance—represents a central, and widely explored mechanism in PD, though it remains poorly explored in melanoma. Evidence on the interaction between α-syn and autophagy in melanoma is limited and somewhat contradictory, mirroring the dual nature of oxidative stress and autophagy themselves, which can act both as stimulatory and detrimental factors for melanoma cell viability [61].

For example, the anti-α-syn-oligomerizing peptide anle138b was shown to promote melanoma cell death in vitro through the dysregulation of autophagy flux, suggesting that advanced melanoma may exploit α-syn to promote autophagy-related cell survival [62]. Instead, in SK-MEL-28 cells, excess α-syn increased cellular resistance to autophagy induction, suggesting that α-syn is a negative modulator of the autophagic tone besides being a cargo of autophagy [118]. The findings of the present study also suggest that possible therapeutic strategies should ideally be implemented before the onset of frank α-syn accumulation to preserve the efficacy of cellular clearance mechanisms. Interestingly, in SK-MEL-28 cells, autophagy modulation and DA exposure were shown to modify the endogenous localization of α-syn between the nucleus and cytoplasm. In detail, while autophagy inhibition and DA exposure both promote cytoplasmic accumulation of α-syn, autophagy induction favors increased nuclear localization of α-syn, underscoring the functional relevance of the subcellular α-syn distribution [118]. Recent evidence documented that the reduction in plasma membrane recycling of adhesion proteins (L1CAM, CD81, TfR1) observed in SNCA-KO SK-MEL-28 cells is associated with an increased density of endolysosomes due to impaired anterograde trafficking and perinuclear clustering [30]. Although the authors did not directly dissect the crosstalk with autophagy-related compartments, this study suggests that α-syn positively modulates the trafficking of sequestration/degradative organelles to support melanoma progression [30].

Compelling insights into a possible connection between α-syn, autophagy dysfunction, and Fe overload stem from studies in retinal epithelial cells (RPE), which similarly to melanocytes and DA neurons, contain melanin and are associated with visual disturbances in age-related diseases, including classical synucleinopathies such as PD [119, 120]. In this case, α-syn was shown to impair ferritinophagy, the autophagy/lysosomal-dependent degradation of ferritin, which results in the accumulation of Fe-rich ferritin in the outer retina in vivo and RPE cells in vitro [76, 121]. The results of this study suggest that Fe overload and impaired ferritinophagy initiate the prion-like spread of α-syn and ferritin, fueling retinal Fe dyshomeostasis and associated cytotoxicity, which deserves to be investigated in the frame of melanoma, and PD-melanoma connection as well.

### DNA damage response

Reports about α-syn localization in the nucleus have steadily increased in recent years, representing a topic of ongoing investigation. In the nucleus, α-syn can interact with nuclear proteins, histones, DNA, and DNA repair proteins to modulate chromatin structure and transcription, as well as Double Strand Breaks (DSB) repair pathways, and cell cycle genes associated with cell differentiation, and signaling [74, 82, 122, 123]. The significance of nuclear α-syn localization in the frame of PD pathobiology remains to be fully elucidated. Mechanistic studies showed that in the nucleus, α-syn interacts with Ras-related nuclear protein (RAN) and normally functions in the nucleocytoplasmic transport [123]. This is instead disrupted by SNCA mutations and triplications resulting in RAN sequestration and impaired transport of nuclear proteins that are key in maintaining normal nuclear function, such as DNMT3A [123]. Furthermore, as Lewy inclusion-containing neurons show increased DSB levels in both experimental mice and human-derived tissue, a model has been proposed whereby cytoplasmic aggregation of α-syn reduces its nuclear levels, and increases DSBs, potentially contributing to programmed cell death via nuclear loss-of-function [82].

**Fig. 3:**
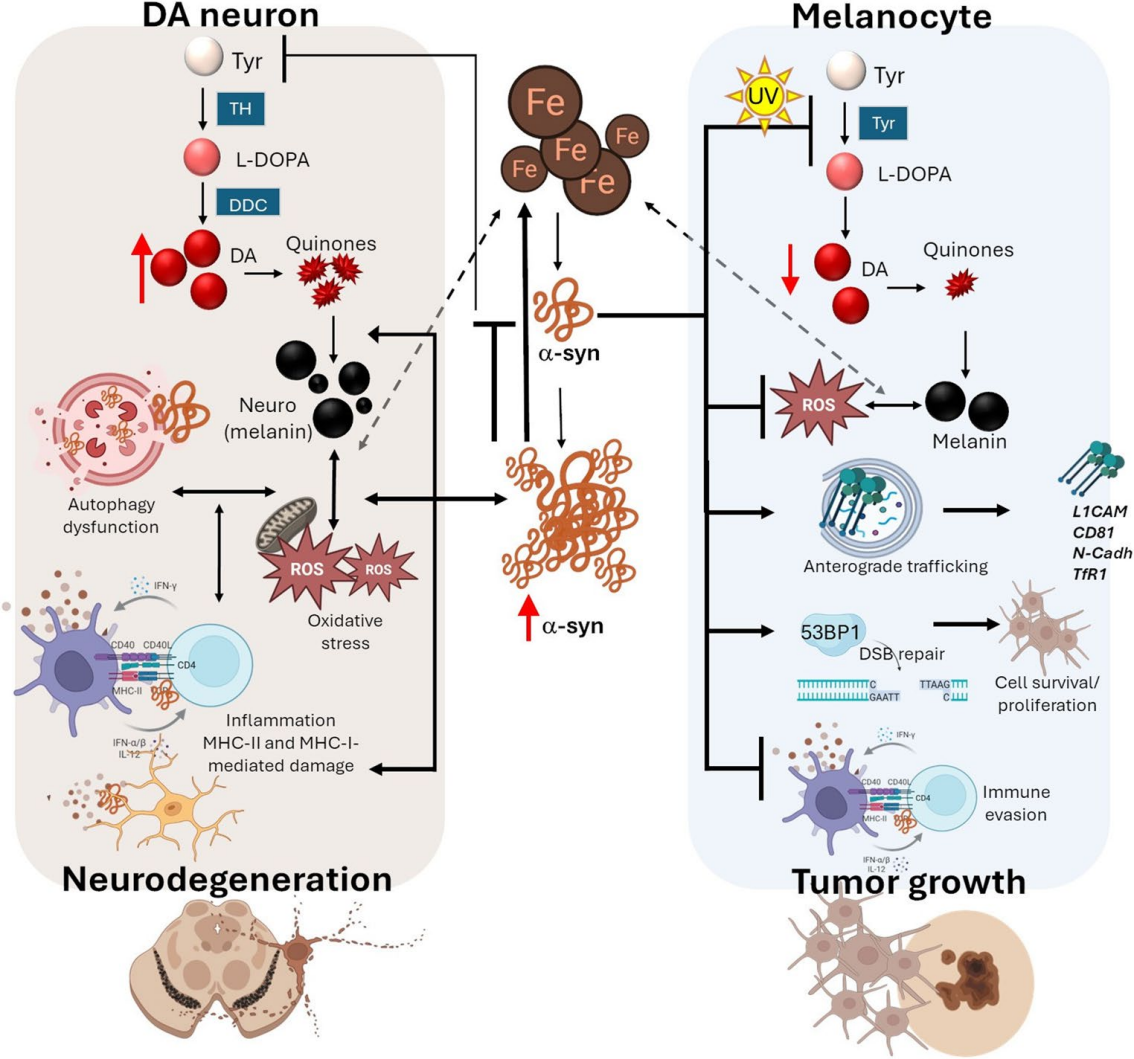
α-Syn-related effects at the crossroad of neurodegeneration and melanoma. Schematic overview of α-syn’s role in neurodegeneration within DAergic neurons (left) and melanocytic malignant transformation (right). Under physiological conditions in DA neurons, α-syn downregulates TH activity to limit DA synthesis. However, altered α-syn proteostasis (accumulation/aggregation), through a loss-of-function mechanism, contributes to increased intracellular DA, and its conversion to toxic quinones and neuro(melanin). In this frame, iron and (neuro)melanin overload could either initiate or exacerbate α-syn accumulation and/or aggregation. In summary, within DAergic neurons, excess α-syn and its aggregation into insoluble species intermingle with a vicious cycle of harmful events, including exacerbation of iron-, DA-, and neuro(melanin)-related oxidative stress, autophagy dysfunction, inflammation, and immune-mediated neuronal damage. In melanocytes, α-syn blunts DA and melanin synthesis, especially under stress conditions (e.g. UV) and promotes iron uptake by increasing TfR1 availability at the plasma membrane. Simultaneously, α-syn restrains potential iron- and melanin-related oxidative stress and promotes DNA damage repair. Moreover, α-syn promotes the anterograde trafficking of key adhesion and migration proteins (L1CAM, CD89, N-Cadherin) to increase their availability at the plasma membrane meanwhile toning down immune responses to promote tumor immune evasion. Collectively, these events promote melanocytic cell survival, proliferation, and migration, thereby supporting melanoma development. The figure was created in part with BioRender (www.biorender.com). Full figure was assembled by the authors

Very recent results from the TG3 mouse model, which spontaneously develops melanoma, showed that SNCA-KO delays melanoma onset and slows tumor growth in association with decreased DNA damage and increased apoptotic markers [73]. Other recent data indicate that α-syn is preferentially enriched within the nucleolus in melanoma cells, where it colocalizes with DSB and the DNA damage repair marker 53BP1 [71]. Specifically, inducing DSB within nucleolar ribosomal DNA (rDNA) increased α-syn levels near sites of damage, and in turn, SNCA-KO increased DNA damage within the nucleolus both at baseline, and after specific rDNA DSB induction, meanwhile prolonging the rate of recovery from this induced damage [71]. These findings suggest that α-syn upregulation in melanoma may be part of a mechanism to improve DSB repair, allowing cells to evade the programmed cell death that would otherwise be triggered by excess genomic damage. A graphical summary of α-syn-related processes in melanoma and PD is provided in Fig. 3 (Fig. 3).

## α-Syn in UV-induced damage: a protective, or risk-modifying role?

Based on the existing evidence discussed, we now turn to an established environmental driver of melanoma, UV radiation. UV is known to be a major risk factor in the etiology of malignant skin melanoma, with an estimated 60–70% being caused by UV exposure. In particular, cumulative exposures to the UVA (315–400 nm) and UVB (280–315 nm) wavelengths of sunlight play a crucial role in inducing biological alterations associated with melanomagenesis, such as DNA damage, oxidative stress, and skin inflammation [124, 125]. There are clearly several factors that determine the skin’s response to UV radiation, including hair color, skin type, genetic background, geographical location and history of tanning.

While most experimental studies focused on UVB exposure to induce experimental melanoma due to its direct DNA-damaging effects, recent evidence has shown that UVA also has a carcinogenic effect [60]. In fact, apart from being more abundant than UVB in natural sunlight (accounting for 95% of solar UV radiation), UVA also penetrates deeper into the dermis, causing damage to the skin and ultimately tumorigenesis via oxidative stress-induced DNA damage. In this context, melanin plays a fundamental, though controversial role, as revealed by a study employing UVA and UVB exposure at biologically relevant doses to induce malignant skin melanoma in mice models [60]. In detail, on the one hand, melanin effectively absorbs and dissipates UVB radiation, reducing direct DNA damage and thereby lowering the risk of UVB-induced skin cancers; on the other hand, under UVA exposure, melanin may act as a photosensitizer, leading to oxidative stress and DNA damage, which increases the risk of melanomagenesis [60]. This duality is reminiscent of that observed for α-syn in melanin biosynthesis. In detail, overexpression of α-syn in melanoma cell lines reduces UVB-induced melanin synthesis, while it contributes to increasing melanin content in DAergic neuronal cells [32]. Again, while no reports exist on α-syn and UVA in relation to melanin synthesis, it was shown that UVA stimulates α-syn increase in association with autophagy dysfunction in fibroblasts [126].

These findings strengthen the hypothesis that α-syn upregulation in melanoma, and the consequent reduction of melanin synthesis may occur as a compensatory mechanism against oxidative damage, which deserves to be investigated in a naturally occurring context of combined UVA + UVB exposure.

Though exposure to light has been rarely considered as a possible risk factor in PD, increasing evidence suggests that it may be pertinent instead [127]. This applies to both artificial and natural light, and obviously, it accounts for the influence of several factors such as age, sunlight intensity, duration, and geographical exposure. For instance, a recent nationwide French ecologic study documented an age-dependent association between UVB and PD incidence [128]. In subjects below the age of 70 years, higher UVB exposure was inversely associated with PD incidence, while lower exposure was associated with higher PD incidence instead, fitting evidence about a correlation between PD and deficiency in vitamin D, levels of which are strictly correlated to UVB exposure. Contrariwise, a positive association between higher light exposure and PD incidence was observed in older subjects [128]. These pieces of evidence underscore the need for further research to explore whether α-syn is implicated in the possible effects of UV exposure in neurodegeneration and abnormal cell proliferation underlying PD-melanoma comorbidity.

## Conclusions

Over the past decades, the possible relationship between PD and melanoma has been strengthened by accumulating epidemiological, clinical and molecular studies. Despite the apparently different cellular outcomes characterizing PD and melanoma—neurodegeneration and uncontrolled proliferation, respectively—the two diseases exhibit common pathogenic mechanisms centered on α-syn accumulation. Altogether, the pieces of evidence herein reviewed suggest a vicious cycle in which iron overload, DA/melanin-mediated redox activity, and immune dysregulations reinforce α-syn accumulation, DNA damage response, and alterations of cell clearance, eventually contributing to neurodegeneration on the one hand, and tumor progression, on the other (Fig. 3). The apparently paradoxical roles of α-syn in melanocytes, melanoma, and DAergic neurons, at times overlapping, and at others opposing (Table 2), underscore its highly- context- and cell-type–specific activities, which are indeed shaped by environmental insults that affect overall cellular proteostasis, the intrinsic proliferative potential and vulnerabilities of the cells involved, as well as the inherent properties of α-syn, including its conformational states, and subcellular localization.

**Table 2:** Expression, localization, and cellular effects of α-syn in melanocytes, and daergic neurons in physiological and pathological conditions

Feature	Normal Melanocytes	Normal DAergic neurons	Melanoma cell	DAergic neurons PD
α-syn/SNCA expression	Physiological, MITF-regulated[18, 38].	Physiological[3].	High: Overexpressed, Phosphorylated, especially in advanced tumor stages [18, 26, 62–64, 66].	High: Overexpressed, Mutated, Phosphorylated, Aggregated [1, 3, 5, 6, 51, 52].
Effect on melanin/DA synthesis	No major effects under basal conditions[38].	Reduces TH activity and tones down DA conversion into neuromelanin.	Reduces UVB-induced upregulation of melanin synthesis via TYR inhibition[32].	Loss of TH-inhibiting activity: increased DA conversion into neuromelanin and toxic melanin precursors.
Role in vesicle trafficking/secretion	Supports melanosome maturation, trafficking, and secretion[38].	Regulates synaptic vesicle trafficking, fusion, and release[3, 42].	Disrupts melanosome trafficking/release[39].	Disrupts vesicle trafficking and release[3, 4].
Effect on cell proliferation, differentiation, and morphology	Promotes melanoblast-to-melanocyte differentiation. Supports dendritic morphology[38].	Reduces cell proliferation and controls the expression of cell cycle genes associated with differentiation, and signaling[74].	Promotes cell survival and proliferation[62, 66, 70, 72, 73]. Promotes cell motility via upregulation/membrane recycling of CD81, L1CAM and N-cadherin[29, 30, 68].	Alters neurite morphology and promotes progressive neuronal degeneration[40, 41].
Subcellular localization	Cytoplasmic, mitochondria-and melanosome-associated, and nuclear[38].	Cytoplasmic, vesicle- and mitochondria-associated, and nuclear[3, 122].	Cytoplasmic, melanosome-associated, nuclear/nucleolar[22, 71].	Cytoplasmic inclusions (Lewy bodies), mitochondrial, nuclear.
Response to oxidative stress and DNA damage response	Not clearly defined.	Scavenger, chaperone-like activity[75]. Involved in histone-interaction, DNA repair[82, 123].	Counteracts oxidative stress, interacts with DJ-1, decreases apoptosis, and promotes DNA damage repair[66, 70, 71, 73].	Fuels oxidative stress. Impairs DNA repair response[82, 123].
Interaction with Fe metabolism	Acts as a ferrireductase and contributes to Fe (II)- melanin binding [112].	Acts as a ferrireductase and contributes to Fe (II)- neuromelanin binding [112].	Increases reactive Fe (II) and modulates TFR-1/ferritin balance[70].	Exacerbates Fe (II) toxicity[77].
Interaction with autophagy	Not clearly defined.	Not clearly defined: autophagy cargo, and possibly modulator?	Not clear: enhances autophagy flux or promotes resistance to autophagy induction[62, 118]. Cytoplasmic-to-nuclear translocation under autophagy induction [118].	Impairs the autophagy-lysosome system[117].
Interaction with the immune system	Not well-characterized, low immunogenic profile under normal conditions.	Prevents neuroinflammation and keeps low basal MHC expression under normal conditions[100].	Supports immune evasion via MHC-II downregulation[65]. Not clear: promotes or suppresses local tumor inflammation[69, 72].	Promotes microglial activation, secretion of pro-inflammatory cytokines/chemokines, T cell-recruitment, MHC-I/II upregulation[80, 99].

Such considerations, coupled with the bulk of data on the evolutionary-conserved expression and pleiotropic functions of α-syn across different cell types and tissues, raise the question of whether α-syn should be considered as a culprit of disease, as a common outcome from shared upstream mechanisms, or finally, as a compensatory response to prevent cellular stress-induced cell death. We believe that α-syn accumulation could, at least in its early phases, represent an adaptive, compensatory response to protect against alterations in DA and (neuro)melanin metabolism, oxidative stress and cellular injury. In this scenario, α-syn upregulation may represent an attempt to preserve cell survival by modulating DA/(neuro)melanin synthesis, redox balance, and DNA repair. In melanoma cells, this protective function is maintained, as the ongoing cell turnover allows the dilution of any potentially harmful, excess, or misfolded α-syn species. In contrast, DAergic neurons, being highly specialized, and post-mitotic cells, lack regenerative capacity and cannot dilute excess or toxic α-syn conformations through division, a limitation that may ultimately render this protective function ineffective. In fact, under chronic insults, including intracellular DA, iron, and neuromelanin overload, overwhelmed cellular clearance fosters α-syn transition from a stress-buffering molecule into a pathogenic species that contributes to a vicious, self-reinforcing cycle of harmful events culminating in neurodegeneration, as observed in PD. While this scenario is increasingly gaining ground in the scientific community [129–131], the observed dichotomy highlights how cell type–specific vulnerabilities and differential stress adaptations may dictate whether α-syn acts as a safeguard or as a mediator of disease.

### Future directions

Addressing whether α-syn accumulation represents a functional driver of disease or an epiphenomenon of upstream stress response, calls for a careful evaluation of possible unintended effects of therapeutic strategies directly targeting α-syn. A critical examination of available evidence instead supports focusing on upstream regulators of α-syn proteostasis, such as iron handling, (neuro)melanin metabolism, and cell-clearance systems as more promising therapeutic targets. Modulating these interconnected systems may prevent abnormal α-syn buildup without interfering with its endogenous/homeostatic functions.

To achieve this, refined experimental models that better capture PD and melanoma comorbidity are essential. In fact, although numerous experimental models exist for both PD and melanoma, including those overexpressing or harboring mutant forms of SNCA/α-syn, they rarely capture the shared environmental, cellular, and immune components that underpin their comorbidity. Notably, there is a paucity of studies investigating melanoma-relevant processes within α-syn-based PD models. Future work should therefore explore α-syn functions in physiologically relevant settings of melanoma development, such as combined UVA + UVB exposure, as well as multicellular systems incorporating fibroblast-keratinocyte-melanocyte and immune cell interactions, alongside longitudinal and cell-type–specific α-syn manipulations. These integrative approaches will enable a dynamic understanding of α-syn’s context-dependent functions throughout disease stages and will contribute to uncovering pathogenic mechanisms and therapeutic vulnerabilities shared by PD and melanoma.

## Data Availability

Data sharing is not applicable to this article as no new data were created.
